# Rapid, CRISPR-Based, Field-Deployable Detection Of White Spot Syndrome Virus In Shrimp

**DOI:** 10.1038/s41598-019-56170-y

**Published:** 2019-12-23

**Authors:** Timothy J. Sullivan, Arun K. Dhar, Roberto Cruz-Flores, Andrea G. Bodnar

**Affiliations:** 1 The Gloucester Marine Genomics Institute, Gloucester, MA 10930 USA; 2 Aquaculture Pathology Laboratory, School of Animal and Comparative Biomedical Sciences, The University of Arizona, Tucson, AZ 85721 USA

**Keywords:** Animal biotechnology, Biological techniques

## Abstract

Rapid, sensitive, point-of-care diagnostics are critical for managing infectious diseases. Here we adapt the CRISPR-based SHERLOCK method to develop a rapid, accurate, single copy detection assay for White Spot Syndrome Virus, the most devastating virus impacting global shrimp aquaculture. Further, we combine paper matrix nucleic acid extraction and lateral flow colorimetric reporting to create a fully field-deployable, next-generation diagnostic with potential to transform veterinary pathology, disease ecology, and animal production.

## Introduction

Mitigating the spread of infectious diseases in agriculture, livestock production, and aquaculture is of paramount importance for global food security. Aquaculture has grown at an incredible pace over the past 50 years^1,2^ and it is predicted that production will need to double its current supply in order to meet demands of the growing human population^1,3,4^. One of the greatest and most complex impediments to aquaculture production is the spread of infectious diseases which pose a significant threat to both capture fisheries and sustainable aquaculture. Availability of rapid, sensitive and cost-effective diagnostics are critical for limiting transmission and mitigating disease outbreaks. However, current diagnostics using histology and conventional and real-time PCR require sophisticated equipment, expensive reagents, are time intensive, and cannot be employed in the field. Therefore, there is an urgent need for new methodologies that are inexpensive and rapid, yet highly sensitive, enabling point-of-care testing in the field particularly in developing and underdeveloped nations where limited infrastructure exists but aquaculture is growing rapidly.

Advances in sequencing and synthetic biology have led to breakthroughs in early disease detection in humans. This is especially true in recent years where isothermal technologies and CRISPR enzymes have enabled “next-generation” diagnostic technologies that are both more sensitive, more portable and easier to use than previous techniques. The SHERLOCK (Sensitive High Efficiency Reporter unLOCKing) method was developed for rapid (~1 hour), sensitive (attomolar) detection of Zika and Dengue, and was successfully converted to lyophilized paper strips for diagnostic testing with no electricity or infrastructure in place^5–7^. There is an urgent need for similar innovation to ensure sustainable growth of aquaculture worldwide. The majority of aquatic animals are cultured in remote areas without access to laboratory facilities. Likewise, many cultured aquatic species suffer from rapidly-developing, high-mortality epizootics that can only be mitigated with enhanced biosecurity, early detection, and rapid action^4^. For most aquaculture species, including shrimp, therapeutics are unavailable and therefore disease prevention through early detection remains the cornerstone of disease management. Here, we document the development of a quick, accurate, and sensitive diagnostic for the detection of White Spot Syndrome Virus (WSSV), the etiologic agent of white spot disease (WSD), the most economically impactful disease infecting shrimp aquaculture worldwide.

The Pacific white shrimp, *Penaeus vannamei*, is the most successful crustacean aquaculture species with a value estimated at $15 billion USD per year^4^ (www.fao.org). However, infectious disease outbreaks routinely impact shrimp farms^8^, resulting in the loss of reared shrimp and limiting both domestic and global production. Principally, these diseases have been viral in nature, from Infectious Hypodermal and Hematopoietic Necrosis Virus (IHHNV; emerged in 1981), Yellow Head Virus (YHV; 1991), Taura Syndrome Virus (TSV;1992), White Spot Syndrome Virus (WSSV; 1992), and Infectious Myonecrosis Virus (IMV; 2004). While some viruses, such as IHHNV, primarily cause growth retardation, others result in varying levels of mortality. For instance, WSSV is so lethal that it can cause fast and large-scale mortality approaching 100% in as little as 1 week.^8,9^. The overall impact of these diseases is best exemplified in their costs for global shrimp production. Estimates range from 100 million to 15 billion dollars per disease and over 20 billion dollars in total^8,10^, resulting in large-scale efforts to develop diagnostic tools to support early detection and mitigate the spread of these pathogens.

White Spot Syndrome Virus is far and above the most influential pathogen of Pacific white shrimp. Since 1992, it has caused over 15 billion dollars in losses^8,10^. Consequently, it has been a focus of diagnostic development for nearly 30 years, including: PCR methods^11^, *in-situ* hybridization^12^, quantitative PCR^13^, Genedrive assays^14^, loop mediated isothermal amplification^15^, monoclonal antibodies^16^, lateral flow immunoassays^17^, sandwich ELISAs^18^, electrochemical detection^19^, and Cas12 assays^20^. Although these techniques solve some aspects of the diagnostic problem, they have a number of limitations. Many of these approaches require sophisticated equipment, electricity, and specialized training. Also, some lack the sensitivity required to be an early detection mechanism. The next generation diagnostic developed here addresses all these issues. By combining isothermal amplification techniques, synthetic biology approaches, and CRISPR detection we have created a WSSV diagnostic with single copy detection that when paired with paper matrix based nucleic acid extraction and lateral flow reporting requires no sophisticated equipment or electricity and is a truly field-deployable, point-of-care diagnostic method.

**Figure 1:**
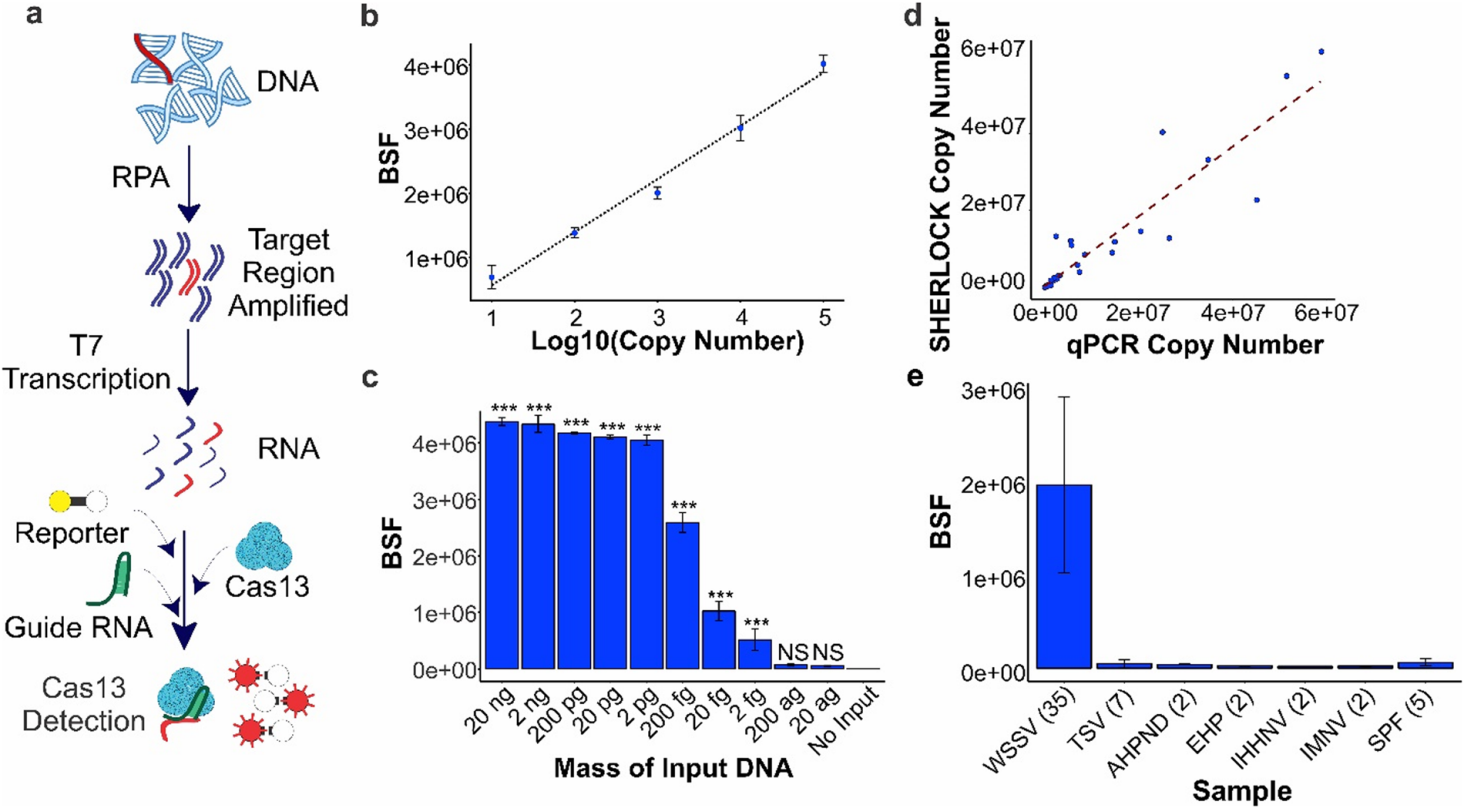
Principles and Performance of our WSSV assay. (**a**) Schematic representation of the SHERLOCK method for nucleic acid detection. (**b**) SHERLOCK assay 10-fold standard curve from 10 to 100,000 copies for the detection of synthetic WSSV viral template. Copy number was Log10 transformed. Equation for line of best fit was: y = 830117 × −268875, *R*^2^ = 0.988. Error bars denote standard deviation. BSF indicates background subtracted fluorescence. (**c**) Limit of detection analysis for the SHERLOCK assay using a diluted positive shrimp sample. Reaction input ranges from 20 ng of DNA to 2 ag of DNA translating to 10,600,000 to 0.001 copies per reaction. NS = not significant, *p < 0.05, ** = *p* < 0.01, ***p < 0.001 based on comparisons to no input control from ANOVA and Tukey’s post-hoc results. Error bars denote standard deviation. BSF indicates background subtracted fluorescence. (**d**) Results of quantification of copy number for WSSV infected white shrimp with both qPCR and SHERLOCK assays indicated a strong correlation: *r* = 0.93 which was significant (*p* = 1.4e^−15^). (**e**) Specificity tests evaluating detection of WSSV with other common white shrimp diseases including TSV, *Vibrio* spp. causing AHPND, EHP, IHHNV, IMNV, as well as SPF shrimp. Numbers next to each name indicate the number of samples tested. Error bars denote standard deviation. BSF indicates background subtracted fluorescence.

In 2017, Gootenberg and colleagues paired the collateral ribonuclease activity of Cas13a with isothermal amplification to create a diagnostic test called SHERLOCK for detection of human pathogens^6^. The SHERLOCK method begins with isothermal amplification through Recombinase Polymerase Amplification, followed by T7 transcription to produce RNA from amplified copies, and lastly Cas13a detection to enable fluorescent or colorimetric reporting (Fig. 1a). Here we detail our development and validation of a new molecular diagnostic for use in the aquaculture industry. By adapting the SHERLOCK method to the detection of WSSV, we have created a molecular tool powered by synthetic biology and CRISPR that can provide point-of-care detection at the pond site.

## Results and Discussion

We designed and developed primers and a guide RNA probe for the detection of WSSV (targeting viral envelope protein 108; *VP108*) infecting the Pacific white shrimp using publicly available genome sequence. As a primary measure of sensitivity and linearity, we generated a 10-fold standard curve by diluting PCR products of synthetic WSSV target DNA regions. This standard curve was able to detect down to 10 copies and showed strong correlation with input DNA (y = 830117 × −268875, *R*^2^ = 0.988, Fig. 1b). We then evaluated the limit of detection of this assay by taking 20 ng of DNA from a single infected sample (*Pvan*005, verified by histopathology) and conducting a 10-fold serial dilution down to 20 attograms of input DNA. The SHERLOCK assay was able to detect down to 2 femtograms of input DNA which based on the copy number of the individual (~530,000 copies/ng DNA) was ~1.06 copies or single copy detection (Fig. 1c). Then, we tested 35 WSSV infected *P. vannamei* samples from experimental challenges with both SHERLOCK and OIE (The World Organization for Animal Health, Paris, France)-recommended qPCR alongside standard curves to quantify and compare WSSV copy number determined by these two methods. Infection was detected in all positive samples and the two approaches showed strong correlation in the copy numbers estimated for each sample (*r* = 0.93, *p* = 1.4e^−15^, Fig. 1d). Lastly, to determine the specificity of our assay, we tested a number of individuals infected with other common shrimp pathogens (Acute Hepatopancreatic Necrosis Diseases [AHPND] caused by *Vibrio parahaemolyticus* producing binary toxin*, Enterocytozoon hepatopenaei* [EHP], IHHNV, IMNV, and TSV) as well as verified Specific Pathogen Free [SPF] *P. vannamei* samples. In all cases, the SHERLOCK assay showed no positive detection (Fig. 1e). In total, these results show our assay is a highly accurate diagnostic, possessing exceptional diagnostic sensitivity, analytical sensitivity, and analytical specificity for use in detecting WSSV infecting marine crustaceans.

**Figure 2:**
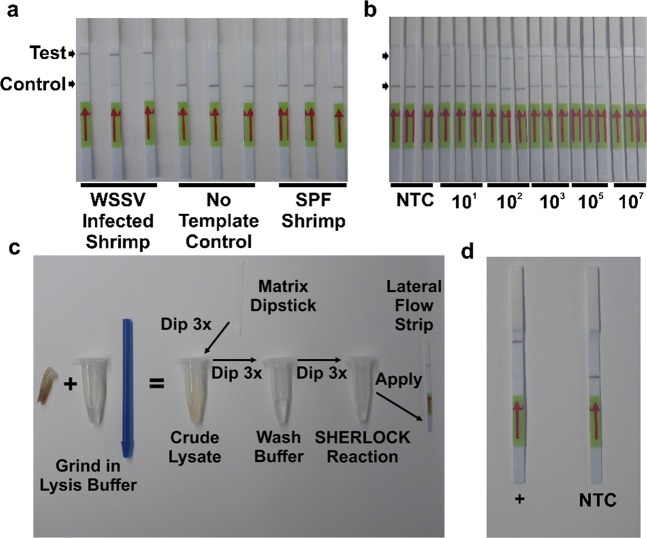
Adapting our WSSV assay for point-of-care testing in the field. (**a**) Converted lateral-flow, strip-based detection of positive WSSV DNA samples as well as no template control reactions and SPF shrimp DNA. (**b**) Lateral-flow strip-based detection of varying synthetic viral copy numbers ranging from 10 copies to 10,000,000 copies. NTC indicates no template control. (**c**) Schematic representation of the combined matrix extraction and lateral-flow SHERLOCK reaction allowing rapid field-deployable diagnostic testing. (**d**) Results of a field-deployable diagnostic assay using experimentally challenged WSSV-infected shrimp tissue with a 1-hour incubation at room temperature showing strong positive signal for an individual with a copy number of 700,000 copies per ng of DNA.

Following the success of our design, development, and laboratory validation we shifted our diagnostic process to create a field-deployable format that does not require any instrumentation. To eliminate the need for electronic detection devices, we adapted our test to a colorimetric, lateral-flow, strip-based output. Using DNA extracted with traditional column kit extractions we found our lateral flow assay accurately detected WSSV-infected experimentally-challenged shrimp and showed no positive detection for No Template Control reactions or SPF shrimp samples (Fig. 2a). By testing diluted synthetic target DNA of known copy number, our lateral flow assay was able to detect as few as 10 synthetic DNA copies (Fig. 2b). Then to remove the sophistication of the DNA extraction, we utilized a method harnessing binding properties of cellulose to selectively bind and elute nucleic acids from crude lysate^21^. By combining this method with our lateral flow assay (Fig. 2c), we were able to extract, amplify and detect viral DNA from experimentally challenged shrimp in approximately 60 minutes (Fig. 2d). In total, though we primarily assessed and validated our assay’s performance (i.e. measured diagnostic sensitivity, analytical sensitivity, and analytical specificity) using a standard column DNA extraction we have shown that by combining its exceptional properties with those of the paper matrix extraction results in a diagnostic method requiring no electricity, heat, advanced training, or specialized equipment. When testing WSSV-infected muscle tissue, this method takes one hour, can operate at room temperature, and is simple enough to be used by aquaculture professionals under field conditions.

Aquaculture production of Pacific white shrimp far exceeds fishery output, even when taking into account large-scale losses from frequent epizootics. While the production of genetically superior (i.e. resistant) and disease free broodstock systems have pushed the growth of shrimp aquaculture it still suffers from setbacks and requires a focus on enhanced biosecurity monitoring and rapid detection and mitigation methodologies^22^. CRISPR diagnostics have the accuracy to detect particular pathogens in complex reaction environments, the sensitivity to detect the very early stages of an infection (which are most critical), and the ease and effectiveness to be a true point-of-care approach that facilitates testing by laypeople at the pond site. This opens the door to both more effective mitigation and targeted sanitation and biosecurity that prevents disease introduction and spread^22,23^. In conclusion, we have shown the incredible power of the SHERLOCK method for diagnostics that go beyond human medicine and have the potential to shape the future of aquaculture, animal production, and veterinary pathology in ways that limit disease, increase sustainability, and maximize production to meet the needs of a growing global population.

## Materials and Methods

### Experimental challenge of *Penaeus vannamei* shrimp using White Spot Syndrome Virus (WSSV) and detection of WSSV with histopathology

Specific Pathogen Free (SPF) *P. vannamei*, originally obtained from a commercial supplier in the US were challenged via oral route by feeding frozen WSSV infected tissue (China isolate-CN 95) at a rate of 5% body weight of the animals in a tank. The first mortalities were noted on day 3 post-infection and the study was terminated at day 6 when the last animal died. The cephalothorax was dissected from representative moribund animals and preserved in Davidson’s AFA fixative for H&E histology (N = 8) and the tail muscle tissue were frozen at −70 °C. Tissues collected from healthy animals served as control.

All the moribund animals showed intra-nuclear basophilic inclusion bodies in hypertrophied nuclei of the cuticular epithelial cells and connective tissue cells that are pathognomonic of WSSV infection (Supplemental Fig. 1). The infection severity was graded as G4 (in a scale of G1-G4, G1 being the lowest and G4 being the highest levels of infection). None of the healthy animals showed any histological alterations characteristics of WSSV infection.

### Detection of WSSV by real-time quantitative PCR (qPCR)

Total genomic DNA from experimentally challenged shrimp samples was extracted with DNeasy spin kits from Qiagen and checked for quantity and quality on a nanodrop spectrophotometer (Thermo Fisher). WSSV was detected and quantified using a TaqMan™ Fast Virus 1-Step Master Mix (Applied Biosystems™) following an OIE recommended protocol^13^ with reactions run on an ABI StepOne Plus system. Each sample was run in duplicate alongside known copy number control samples for a standard curve from 10–100,000,000 copies.

### Development of the SHERLOCK method for detection of WSSV

All publicly available whole genome sequences for WSSV strains were downloaded as Fasta files (.fas) from GenBank (https://www.ncbi.nlm.nih.gov/genbank/). These sequences included accession numbers: MG264599.1, KT995470.1, AF332093.1, KR083866.1, KT995471.1, KT995472.1, AF440570.1, JX515788.1, KX686117.1, MG702567.1. These genomes were aligned with MAFFT^24^ (Multiple Alignment by Fast Fournier Transformation; mafft.cbrc.jp) using default conditions. We then manually surveyed the length of the genomes for regions that were free from polymorphism and at least 300 bp in length. These genome regions were put into NuPack (www.nupack.org), which estimated RNA structure from this DNA sequence using default conditions at 37 °C. This was done to ensure the RNA product would be free of 3D structure (i.e. bends and hairpins) ensuring it would be open for binding by the guide RNA. For sequences free from structure, we designed 5–10 possible guide RNAs following the criteria outlined in previous studies with humans^5–7^ (Supplemental Table 1). Likewise, we also made sure that these molecules would show limited structuring under the same conditions with NuPack. Lastly, we used the primer design software Primer3^25^ (www.bioinformatics.nl/cgi-bin/primer3plus/primer3plus.cgi) to design primers for RPA following conditions from previous human studies^5–7^. Each forward primer contained a T7 transcription site located directly upstream to allow for generation of RNA from DNA copies. We designed 3–4 pairs of primers surrounding each potential guide RNA region with a sequence length of ~100–150 bp total (Supplemental Table 1). To generate viral templates for testing, we synthesized the target regions spanned by the RPA primers, and added primer regions to the 5′ and 3′ ends to facilitate production of high copy amounts. We also included a T7 transcription site upstream (5′) of the target sequence to allow for generating RNA as necessary (Supplemental Table 1). All gene regions (for testing), guide RNA sequences and RPA primers were ordered as oligonucleotides from IDT (www.idtdna.com).

CRISPR guide RNAs and viral template DNA were converted to RNA by a two-step process. In the first step, synthetic DNA copies were combined with T7 annealing primers, T4 ligase buffer, and water. This reaction mix was heated to 95 °C and then ramped down to 10 °C at 5 °C per minute. Then, in the second step, 3 ul of this template was used as input into Ampliscribe T7 flash transcription reactions (Lucigen) following standard default reaction mix and conditions. After the reaction was completed, we used an RNA clean and condense reaction kit (Zymo) to purify *in vitro* transcribed RNA and subsequently treat with DNase I. These samples were then quantified on a nanodrop spectrophotometer and frozen at −80 °C. Once RNA was isolated, we tested potential assay components by establishing SHERLOCK reactions with RNA input and varying the guide RNA used to determine which guide RNAs bound successfully (Supplemental Fig. 2). Then we tested RPA primers which surround the successful guide RNAs with amplification success evaluated by electrophoresis (Supplemental Fig. 3). Lastly, we combined RPA (using successful primers) with transcription and SHERLOCK reactions (with successful guide RNAs) following the reaction mix in Supplemental Table 2 on a SpectraMax ID3 with a 3-hour kinetic fluorescent run at 37 °C measuring from the bottom of the Corning 384 well plate every 5 minutes with mixing before and between readings. Based on these results, primers WSSV-171350F and WSSV-171503R with crRNA-585 (Supplementary Table 1) were chosen as the most successful assay for continued development. This assay amplifies and detects a 153 bp fragment of WSSV envelope protein 108; *VP108*. It should be noted that this target was chosen over other potential genes simply because its sequence met the physical conditions necessary for assay design outlined in the original descriptions of the SHERLOCK method^5–7^.

### Generating a standard curve for detecting WSSV by the SHERLOCK method

To generate high concentration viral template solutions we ran PCR reactions with our synthetic viral template (from IDT) following a 20 µl Phusion PCR mix (New England Biolabs) using the primers AMPF and AMPR (Supplemental Table 1) and a PCR protocol that included 2 minutes at 94 °C, 35 cycles of 94 °C − 10 sec, 56 °C − 30 sec, 72 °C − 45 sec, with a 5 minute elongation at 72 °C. These products were run on a 2% agarose gel with 1X TAE buffer, visualized on a GelDoc UV transluminator (BioRad), excised, and purified by Qiagen gel purification. These purified copies were evaluated on a Nanodrop spectrophotometer (Thermo Fisher) to determine concentration, which was used to estimate copy number using the NEB copy number converter (nebiocalculator.neb.com). We then diluted this stock down to establish a standard curve from 10–100,000 copies which was run using the SHERLOCK assay (reaction mix in Supplemental Table 2) on a SpectraMax ID3 with a 90-minute kinetic fluorescent run at 37 °C measuring from the bottom of the Corning 384 well plate every 5 minutes with mixing before and between readings.

### Determining the limit of detection of the SHERLOCK assay

After our standard curve experiments, we selected a single individual (*Pvan*005) which had a very high copy number (530,000 copies per nanogram) and serially diluted this sample (with 2 ng/µl salmon sperm DNA) to establish a standard curve of sample DNA with an input mass ranging from 20 nanograms (2e^−8^ grams) to 20 attograms (2e^−17^grams). Each point in this standard curve was run in triplicate for 45 minutes following the SHERLOCK assay conditions outlined previously. To determine the limit of detection, we used an ANOVA model in R 3.2.4 (www.r-project.org) with Tukey’s posthoc tests to compare the no input control fluorescence to each point in the standard curve with significance determining the limit of sensitivity based on input mass (Fig. 1c; Supplemental File 1). Lastly, we translated this mass to copy number based on the quantification of the sample (*Pvan*005–530,000 copies/ng of DNA).

### Detection of WSSV by the SHERLOCK method

Thirty-five Pacific white shrimp samples with known WSSV infection verified by histology and qPCR (from above) were run in triplicate for 45 minutes alongside a standard curve created with synthetic DNA ranging from 1,000,000,000 to 10 copies. We used these results to verify detection of samples by our SHERLOCK assay and to quantify these unknown samples based on our standard curve results (Fig. 1d). We compared our quantification results to those generated by qPCR using correlative statistics in R 3.2.4 (www.r-project.org; Supplemental File 2).

### Determining the specificity of the SHERLOCK assay

SHERLOCK reactions were established to test the specificity of our assay against a suite of other common shrimp aquaculture pathogens including: Taura Syndrome Virus, *V. parahaemaolyticus* causing Acute Hepatopancreatic Necrosis Disease, *Enterocytozoon hepatopenaei*, Infectious Hypodermal and Hepatopoetic Necrosis Virus, and Infectious Myonecrosis Virus as well as against Specific Pathogen Free shrimp with No Template Controls. Each sample was run in triplicate with a 45-minute kinetic fluorescent run at 37 °C measuring from the bottom of the Corning 384 well plate every 5 minutes with mixing before and between reads to determine whether or not the samples resulted in positive fluorescence readings indicating our assay detected viral template in those samples (Fig. 1e).

### Converting to colorimetric lateral flow format

To make our assay field-deployable, we converted the reporting system from fluorescence to colorimetric reporting. To do this, we ordered lateral flow “hybridetect” testing strips from Milenia Biotec (https://www.milenia-biotec.de/en/) available through TwistDX (twistdx.co.uk) and ordered a custom reporter for use in colorimetric reactions. This reporter was a Fam-PolyUracil-Biotin reporter comprised of 10 Uracil molecules. This allowed us to take advantage of the FITC-antibody-gold nanoparticle chemistry of the Milenia strips to bind the biotin in both cleaved and non-cleaved reporters from the SHERLOCK reaction producing horizontal lines to indicate the presence of reporter (control band) and the presence of the target (cleaved reporter; test band). All of the other reaction conditions were identical to those outlined previously. At the end of the reaction, 20 µl of reaction solution was mixed with 100 µl of detection buffer and a lateral flow strip was added to the tube for 5 minutes. Then the presence of bands was scored. To test this system, we first ran 3 samples in triplicate SHERLOCK reactions: a positive shrimp DNA sample, a no template control, and a specific pathogen free DNA sample with a 45-minute incubation at 37 °C (Fig. 2a). Then we ran triplicate reactions using the copy number solutions prepared previously to evaluate sensitivity of the lateral flow assay. For these reactions we used solutions of 10, 100, 1000, 100000, and 10000000 copies and also incubated for 45 minutes before application to the lateral flow strip.

### Testing the paper matrix nucleic acid extraction

To eliminate the need for complex equipment and facilitate DNA extraction, we utilized a method that takes advantage of the binding properties of cellulose fibers^21^. We first prepared matrix dipsticks by dipping Whatman #1 filter paper in melted Paraplast (Sigma)^21^. This was hung to dry and then cut into thin strips. To test this method, we used two different possible extraction lysis buffers: (1) 20 mM Tris [pH 8.0], 25 mM NaCl, 2.5 mM EDTA, and 0.05% SDS and (2) 1.5 M Guanidine Hydrochloride, 50 mM Tris [pH 8.0], 100 mM NaCl, 5 mM EDTA, and 1% Tween-20. The extraction method involved grinding 2 mg of muscle tissue in lysis buffer, shaking the lysate, dipping the dipstick in the lysate 3 times, dipping the dipstick in wash buffer (10 mM Tris, 0.01% Tween-20) 3 times, and dipping the dipstick in the SHERLOCK reaction (following reaction mix outlined previously) 3 times (Fig. 2c). This was then incubated for 45 minutes. We ran this extraction-SHERLOCK reaction experiment with both extraction buffers using 2 mg of muscle tissue from a positive experimentally challenged shrimp alongside no template controls with both showing positive detection but stronger signal for the SDS based lysis buffer (Supplemental Fig. 1). This buffer was used in all subsequent reactions.

## Supplementary information

Supplementary Information

## Data Availability

The datasets generated and/or analyzed during the current study are available in either the supplemental files to this manuscript or from the corresponding author upon request.
