# Supplementary material for: Rapid, CRISPR-Based, Field-Deployable Detection Of White Spot Syndrome Virus In Shrimp
Source: Sci Rep. 2019 Dec 23;9:19702. doi: 10.1038/s41598-019-56170-y (PMC6928230; doi:10.1038/s41598-019-56170-y)
Supplement: Supplementary file 1 — Supplementary Information [file 41598_2019_56170_MOESM1_ESM.pdf]

RAPID, CRISPR-BASED, FIELD-DEPLOYABLE DETECTION OF WHITE SPOT SYNDROME VIRUS IN SHRIMP

SUPPLEMENTAL MATERIALS

Timothy J. Sullivan[1*], Arun K. Dhar[2], Roberto Cruz-Florez[2], Andrea G. Bodnar[1]

1 Gloucester Marine Genomics Institute, Gloucester MA, 10930

2 The University of Arizona Aquaculture Pathology Laboratory, Tucson AZ, 85721

*Phone:978-879-4575; email: tim.sullivan@gmgi.org

Supplementary Figure 1. White spot syndrome virus infected subcuticular epithelial cells. (A) Intranuclear pale basophilic inclusion bodies (arrows) typical of WSSV infection (40x). (B) High magnification of the infected cell showing the pale basophilic inclusion bodies (arrows) (100x).

Supplementary Figure 2. SHERLOCK fluorescent output used to determine whether different crRNAs (from Supplementary Table 1) successfully bound and activated the Cas13a enzyme. Only those that showed positive output are plotted.

Supplementary Figure 3. Agarose gel electrophoresis with results of recombinase polymerase amplification for each pair of WSSV RPA primers (from Supplementary Table 1). The only manipulation made to this image is a black/white transformation made during image capture. Lane 1 = WSSV-171340F and WSSV-171487R, Lane 2 = WSSV-171350F and WSSV-171487R, Lane 3 = WSSV-171378F and WSSV-171487R, Lane 4 = WSSV-171340F and WSSV-171503R, Lane 5 = WSSV-171350F and WSSV-171503R, Lane 6 = WSSV-171378F and WSSV-171503R, Lane 7 = WSSV-171457F and WSSV-171580R, Lane 8 = WSSV-171480F and WSSV-171580R, Lane 9 = WSSV-171472F and WSSV-171580R, Lane 10 = WSSV-171457F and WSSV-171587R, Lane 11 = WSSV-171480F and WSSV-171587R, Lane 12 = WSSV-171472F and WSSV-171587R, Lane 13 = WSSV-171457F and WSSV-171619R, Lane 14 = WSSV-171480F and WSSV-171619R, and Lane 15 = WSSV-171472F and WSSV-171619R. Gel was 2% agarose with 1X TAE run for 1.5 hours at 100 volts with SyBr Safe. Red star indicates the chosen primer pair used in developing this assay.

Supplementary Figure 4. Results of field deployable paper matrix extraction & SHERLOCK assay test for both possible lysis buffers. Results showed both lysis buffers resulted in positive detection for infected samples and no amplification for no template control samples. Signal intensity was higher for the “SDS” lysis buffer and this buffer was used for further tests.

Supplementary Figure 1

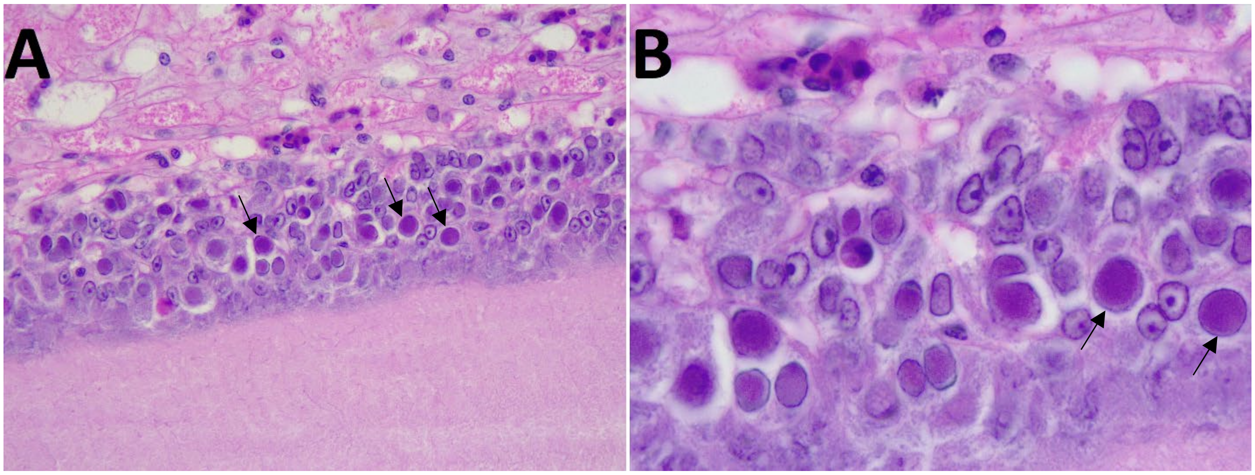

Supplementary Figure 2

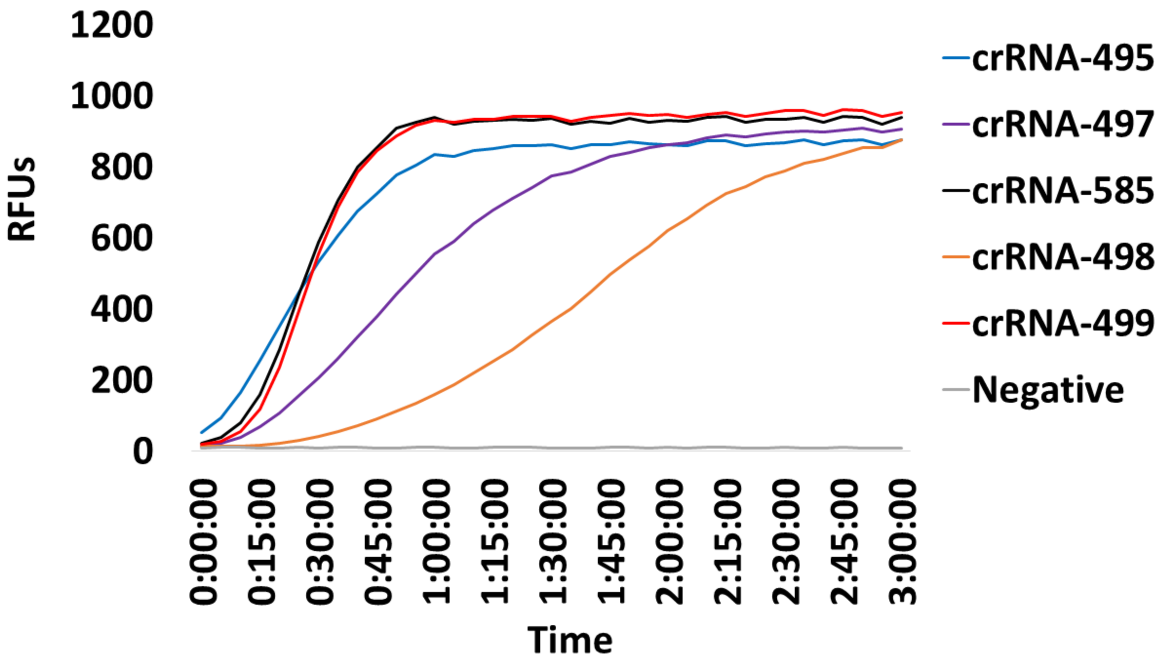

Supplementary Figure 3

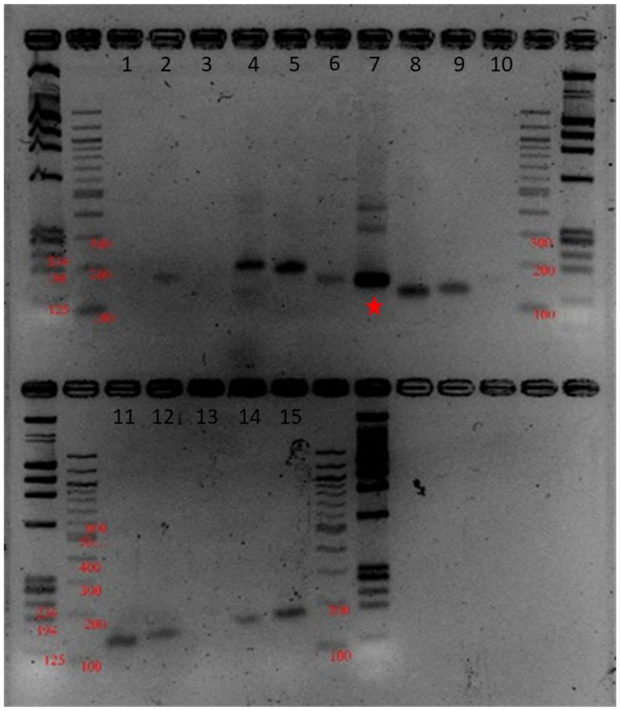

Supplementary Figure 4

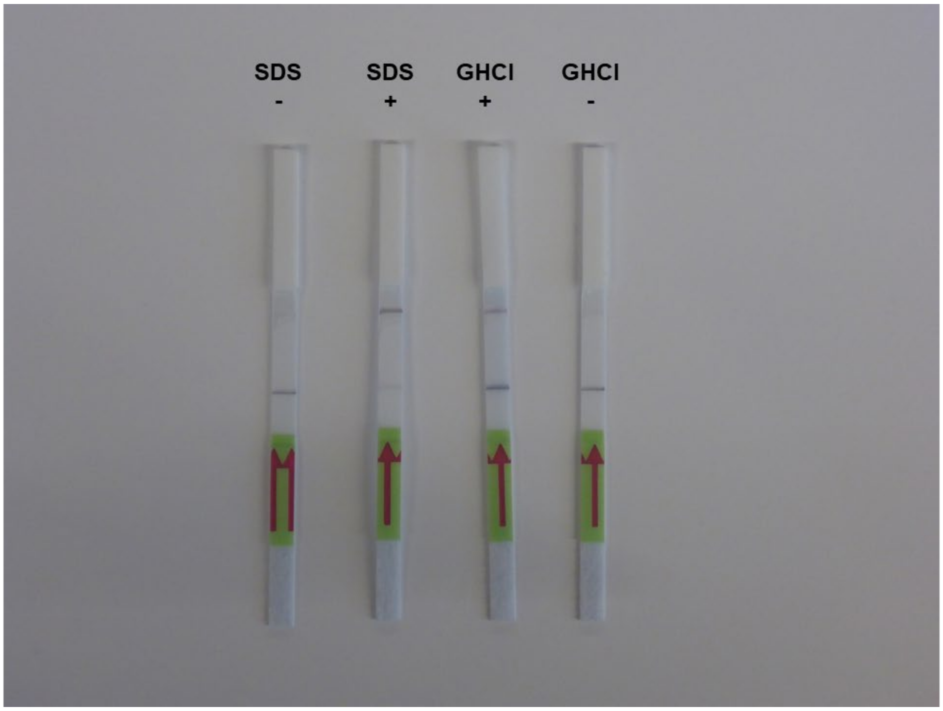

Supplemental Table 1. Sequence for all primers, probes, reporters, targets, and other oligos used in this manuscript with the name of the oligo (Name), the Figures of which the oligo was used (Used in), the sequence (Sequence), and the reference where the oligo comes from (Reference).

Name	Used in	Sequence	Reference
WSSV-crRNA-494	Fig. S2	catataTAATACGACTCACTATAGGGCCACCCCAATATCGAAGGGGACTAAAACgaattcctttccttcgtttctgacggaa	This Study
WSSV-crRNA-495	Fig. S2	catataTAATACGACTCACTATAGGGCCACCCCAATATCGAAGGGGACTAAAACgctaaatcatgactttcgaaccgagaat	This Study
WSSV-crRNA-496	Fig. S2	catataTAATACGACTCACTATAGGGCCACCCCAATATCGAAGGGGACTAAAACtcgaaccgagaattcctttccttcgttt	This Study
WSSV-crRNA-497	Fig. S2	catataTAATACGACTCACTATAGGGCCACCCCAATATCGAAGGGGACTAAAACtgactttcgaaccgagaattcctttcct	This Study
WSSV-crRNA-498	Fig. S2	catataTAATACGACTCACTATAGGGCCACCCCAATATCGAAGGGGACTAAAACaattgcttgcatcttccaccccgtcatg	This Study
WSSV-crRNA-499	Fig. S2	catataTAATACGACTCACTATAGGGCCACCCCAATATCGAAGGGGACTAAAACgaggtgggtaattgcttgcatcttccac	This Study
WSSV-crRNA-579	Fig. S2	catataTAATACGACTCACTATAGGGCCACCCCAATATCGAAGGGGACTAAAACattgcttgcatcttccaccccgtcatgc	This Study
WSSV-crRNA-580	Fig. S2	catataTAATACGACTCACTATAGGGCCACCCCAATATCGAAGGGGACTAAAACggggtgaggtgggtaattgcttgcatct	This Study
WSSV-crRNA-581	Fig. S2	catataTAATACGACTCACTATAGGGCCACCCCAATATCGAAGGGGACTAAAACcagggttagctccttgaactgctttcaa	This Study
WSSV-crRNA-582	Fig. S2	catataTAATACGACTCACTATAGGGCCACCCCAATATCGAAGGGGACTAAAACagctccttgaactgctttcaatttactt	This Study
WSSV-crRNA-583	Fig. S2	catataTAATACGACTCACTATAGGGCCACCCCAATATCGAAGGGGACTAAAACgggttagctccttgaactgctttcaatt	This Study
WSSV-crRNA-584	Fig. S2	catataTAATACGACTCACTATAGGGCCACCCCAATATCGAAGGGGACTAAAACcctttccttcgtttctgacggaacgttc	This Study
WSSV-crRNA-585	Fig. S2	catataTAATACGACTCACTATAGGGCCACCCCAATATCGAAGGGGACTAAAACgacggaacgttccatttattccctttaa	This Study
WSSV-171340F	Fig. S3	gaaattaatacgactcactatagggCGATGGTGGCCTCACTCAATCTATCTAT	This Study
WSSV-171487R	Fig. S3	TTGACTGTTAAAGGGAATAAATGGAACG	This Study
WSSV-171350F	Fig. S3	gaaattaatacgactcactatagggCTCACTCAATCTATCTATATGGTGTTCAGG	This Study
WSSV-171378F	Fig. S3	gaaattaatacgactcactatagggGGCACGAGAAGAGATGAGGATCTATTTG	This Study
WSSV171340F	Fig. S3	gaaattaatacgactcactatagggCGATGGTGGCCTCACTCAATCTATCTAT	This Study
WSSV171503R	Fig. S3	CCTGGACATGATGAATTTGACTGTTAAAGG	This Study
WSSV-171457F	Fig. S3	gaaattaatacgactcactatagggGAACGTTCCATTTATTCCCTTTAACAGTC	This Study
WSSV-171480F	Fig. S3	gaaattaatacgactcactatagggACAGTCAAATTCATCATGTCCAGGAGAG	This Study
WSSV-171580R	Fig. S3	CTAGAAGAATACCTAGAAGCCAGAGGTAG	This Study
WSSV-171472F	Fig. S3	gaaattaatacgactcactatagggTCCCTTTAACAGTCAAATTCATCATGTC	This Study
WSSV-171587R	Fig. S3	AAAGACACTAGAAGAATACCTAGAAGCC	This Study
WSSV-171619R	Fig. S3	AAATTGAAAGCAGTTCAAGGAGCTAACC	This Study
WSSV-gBlock-171311-171718	Fig. 1, Fig. 2, Fig. S2, Fig. S3	acaGGCAATTCCGACGTCTAAGAAACCATCATGACATTAACCTATAAAAATAGGAGtaatacgactcactatagggacagcatctagatatttgttagatttttcgatggtggcctcactcaatctatctatatggtgttcaggcacgagaagagatgaggatctatttgcagctaaatcatgactttcgaaccgagaattcctttccttcgtttctgacggaacgttccatttattccctttaacagtcaaattcatcatgtccaggagagcggggtgaggtgggtaattgcttgcatcttccaccccgtcatgcctacctctggcttctaggtattcttctagtgtcttttcagggttagctccttgaactgctttcaatttacttttcatccctaattcgtgtatagcttgaaaattaacagacattggtttggctacattataggccattgcagcgcagactagcgcaaaaatacagcctaacacgaacacggtcacgaataacgttatttgcgcggtgaagcgcatacttcggttggtagggtaaagtaacggatctcttcgtcgttcatccatcgttcctgatattacaatggatatcttcctagataaatctcatacaggtgccaccctttttTTTTGCTGTTGTTTGTCGGTGAACGCTCTCCTGAGTAGGACAAATCCGCCGCCCTAGACCTAGGGTACtata	This Study
FAM-PolyU-BTN	Fig. 2, Fig. S4	/56-FAM/UUUUUUUUUU/3Bio/	Myhrvold, C. et al. Science. 360, 444-448 (2018).
T7-anneal	Fig. S2	catataTAATACGACTCACTATAGGG	This study
AMP-F	Fig. 1, Fig. S2, Fig. S3	CAATTCCGACGTCTAAGAAACCA	This study
AMP-R	Fig. 1, Fig. S2, Fig. S3	CCTAGGTCTAGGGCGGC	This study

Supplemental Table 2. Reagents, concentrations, and proportions for SHERLOCK reactions used in the present manuscript including the reagent used (Reagent), the concentration (Stock Concentration), and the Volume used in microliters (Volume (μL)).

**Reagent**	**Stock Concentration**	**Volume (μL)**
Forward Primer	10 uM	2
Reverse Primer	10 uM	2
RPA Reaction Buffer	10X	13
dNTPs	10 uM	1.8
RPA E-mix	10X	3
RPA Core Mix	20X	1.75
MgOAc	280 mM	1.75
MilliQ H20	--	4
Cleavage Buffer	10X	3.5
MgCl2+	50 mM	5
DTT	100 mM	0.3
ATP	100 mM	0.3
UTP	100 mM	0.3
CTP	100 mM	0.3
GTP	100 mM	0.3
Murine Rnase Inhibitor	40 U/ul	0.75
Rnase Alert	1 uM	4
T7 polymerase	--	0.6
Cas13a	1.805 uM	0.6
crRNA	30 uM	0.6
**Total**		**45.55**
